# The Efficacy and Effectiveness of Education for Preventing and Treating Non-Specific Low Back Pain in the Hispanic Cultural Setting: A Systematic Review

**DOI:** 10.3390/ijerph19020825

**Published:** 2022-01-12

**Authors:** Francisco M. Kovacs, Natalia Burgos-Alonso, Ana María Martín-Nogueras, Jesús Seco-Calvo

**Affiliations:** 1 Kovacs Back Pain Unit, HLA-Moncloa University Hospital, 81, Avenida Valladolid, 28008 Madrid, Spain; fmkovacs@kovacs.org; 2 Spanish Back Pain Research Network, 28008 Madrid, Spain; natalia.burgos@ehu.es (N.B.-A.); anamar@usal.es (A.M.M.-N.); 3 Departamento de Medicina Preventiva y Salud Pública, Universidad de El País Vasco (UPV/EHU), Campus Universitario, Bº Sarriena s/n, 48940 Leioa, Spain; 4 Department of Nursing and Physiotherapy, University of Salamanca, Avenue Donantes de Sangre s/n, 37007 Salamanca, Spain; 5 Institute of Biomedicine (IBIOMED), University of León, 24071 León, Spain; 6 Visiting Researcher, University of the Basque Country, 48049 Leioa, Spain

**Keywords:** non-specific low back pain, education programs, systematic review, Hispanic cultural setting

## Abstract

A systematic review was conducted to assess the efficacy and effectiveness of education programs to prevent and treat low back pain (LBP) in the Hispanic cultural setting. Electronic and manual searches identified 1148 unique references. Nine randomized clinical trials (RCTs) were included in this review. Methodological quality assessment and data extraction followed the recommendations from the Cochrane Back Pain Review Group. Education programs which were assessed focused on active management (3 studies), postural hygiene (7), exercise (4) and pain neurophysiology (1). Comparators were no intervention, usual care, exercise, other types of education, and different combinations of these procedures. Five RCTs had a low risk of bias. Results show that: (a) education programs in the school setting can transmit potentially useful knowledge for LBP prevention and (b) education programs for patients with LBP improve the outcomes of usual care, especially in terms of disability. Education on pain neurophysiology improves the results of education on exercise, and education on active management is more effective than “sham” education and education on postural hygiene. Future studies should assess the comparative or summatory effects of education on exercise, education on pain neurophysiology and education on active management, as well as explore their efficiency.

## 1. Introduction

“Common” or “non-specific” low back pain (LBP) is defined as pain between the costal margins and the inferior gluteal folds, which is usually accompanied by painful limitation of movement, may be associated with pain referred down to the leg (“leg pain”), and is not related to fracture, direct trauma or systemic diseases, such as neoplastic, infectious, vascular, metabolic, or endocrine-related processes [1,2]. It represents a major health and social burden [1,2,3].

Patient education is recommended to empower patients, improve disability, reduce anxiety, shorten duration of LBP, and reduce the risk of further recurrences [4,5,6,7]. Educational contents and methodology vary significantly across different educational programs, but most address some cognitive and psychosocial aspects assumed to influence disability, prognosis and/or return to work [8,9,10,11,12,13,14,15].

Previous studies have shown that the importance of some psychosocial factors varies across cultural settings. For instance, catastrophism or fear avoidance beliefs (FABs) appear to play a relevant role in the Scandinavian and Anglo-Saxon cultural environments [8,9,10,11,12,13,14,15], while they are irrelevant in the Hispanic cultural environments [16,17,18,19,20,21]. This suggests that the optimal educational strategy for patients suffering from LBP may vary from one cultural setting to another.

As a result, identifying which education programs have been shown to be effective in a cultural setting, and analyzing their comparative effectiveness, is a prerequisite for deciding which one should be implemented in that specific setting.

Therefore, the objectives of this study were to systematically review the available evidence on: (a) the educational programs for preventing or treating LBP which have been assessed in the Hispanic cultural setting and (b) the comparative efficacy and effectiveness of these programs in this specific setting.

## 2. Materials and Methods

This systematic review followed the recommendations from the PRISMA statement [22] and its protocol was registered in the PROSPERO database (CRD 42021236104).

### 2.1. Search and Study Selection

The following electronic databases were searched from inception until 20 September 2021: The Cochrane Library Plus (CENTRAL, Cochrane Systematic Reviews Database), MEDLINE, PREMEDLINE, NHS CRD (DARE, HTA), SCOPUS, Science Citation Index, EMBASE, PEDRO, CINAHL, Current Contents, EMBASE, Family health database, FSTA [Food Science and Technology Abstracts], ISI Web of Knowledge, LILACS, NNNConsult, OvidMD, ProQuest Central, PubMed, SciFinder Scholar, Science Direct, SPORTDiscus, Web of Science, Wiley Online Library, EBSCO Online, Educational Research Abstracts online (ERA), Electronic Library For Social Care, Ergonomics Abstracts, Psych Info/Psych Lit/Psych Abstracts, RECAL Bibliographic Database, Social Science Citation Index, Sociological Abstracts, SCIELO, http://www.clinicaltrials.gov, http://controlled-trials.com, http://www.who.int/ictrp/en/, http://www.ensaiosclinicos.gov.br/,http://isrctn.org/, http://registroclinico.sld.cu/, http://informationr.net/ir/7-1/spanish2.html, and http://www.cindoc.csic.es/basescsic/bibydocinf.html. (accessed date: 20 September 2021).

The search strategy was designed to ensure maximum sensitivity and is shown in Appendix A. No date or language restrictions were applied.

The authors listed the scientific journals they considered most likely to publish RCTs on education for LBP prevention or treatment. These journals were: Spine, Spine Journal, Pain, Clin J Pain, Eur J Pain, Health Sci Inst, Health Promot Perspect, J Sch Health, J Nurs Educ Pract, and BMC Musculoskeletal Diseases. The authors conducted a manual search in the website pages of these journals, in order to assess the comprehensiveness of the references retrieved through the automatic search.

Additionally, references included in the reviewed studies, and in previous systematic reviews focusing on patient education for preventing or treating LBP, were revised to identify additional studies.

Studies were included in this review if they complied with all of the following inclusion criteria:

(a) Design: Randomized controlled clinical trials (RCTs).
(b) Study population: Spanish-speaking, healthy subjects (for studies on prevention) or subjects with LBP, without any age limits. Studies including Spanish-speaking and non-Spanish speaking participants, and studies including participants with and without pain, or with LBP and with other conditions, could be included only when data had been analyzed separately.
(c) Sample size: ≥10 subjects per group must have completed the study.
(d) Interventions: ≥1 group must have received education of any type.
(e) Comparators: natural history, placebo, sham or any health technology intended to prevent or treat LBP (including other types of education).
(f) Outcomes: ≥1 of following outcomes had to have been assessed: pain severity (LBP or referred pain), LBP-related disability, health-related quality of life, knowledge (on strategies to prevent or treat LBP.
(g) Location: studies conducted in any country in which Spanish can be used in Governmental documents and to communicate with the Administration.

References identified through the electronic search were screened based on title and abstract by two authors separately, out of a pool of three (NB-A, AMM-N, and JS-C). The full texts of those which were eligible were assessed for inclusion criteria by two authors separately, out of a pool of three (NB-A, AMM-N, and JS-C). Disagreements on eligibility were resolved by consensus with a fourth author (FMK).

In cases where an aspect of an original study required clarification, the corresponding authors were contacted by e-mail. When the authors were not responsive, two follow-up e-mails were sent at 14 day intervals.

### 2.2. Data Collection Process, Quality Assessment and Data Analysis

The methodological quality of the studies included in this review was assessed separately by two reviewers out of a pool of three (NB-A, AMM-N, and JS-C), and disagreements were solved by consensus with another author (FMK).

Following the recommendations from the Cochrane Back Review Group for assessing the risk of bias [23,24] the methodological quality of each RCT was assessed according to a set of 13 criteria. A study was categorized as “low risk of bias” when it met ≥ 6 of these criteria, although studies with serious flaws were categorized as “high risk of bias” regardless of score [24].

All the key information was extracted and inserted into two tables. The first table contained data on the methodological characteristics (study design, setting, follow-up period, number of subjects included, age of participants, interventions, and statistical analysis). The second table focused on outcome measure and results.

Data extraction was undertaken separately and in duplicate by two authors out of a pool of three (NB-A, AMM-N, and JS-C), using standardized electronic forms. All data on all the variables gathered in each individual study were extracted. The information was summarized through a qualitative synthesis.

No researcher participated in the selection and quality assessment processes of any study he or she had authored.

## 3. Results

**Figure 1:**
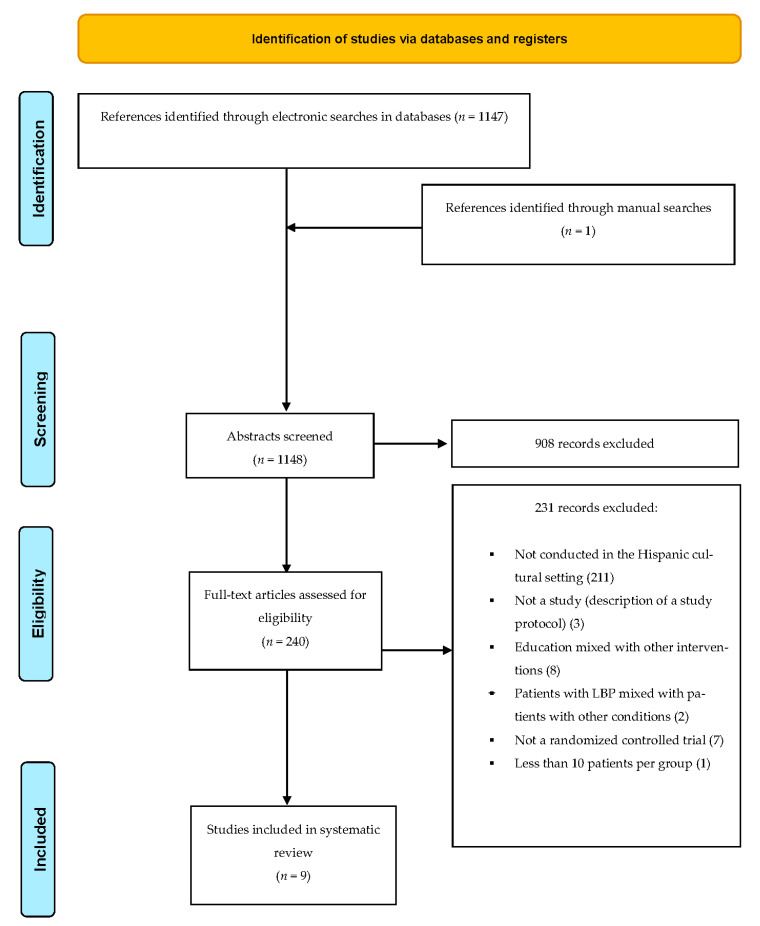
PRISMA Flow Diagram of This Study.

The electronic and manual searches identified 1622 references, 474 of which were duplicates. Among the 1148 unique references, 908 were excluded based on their title and abstract. The full texts of the remaining 240 were assessed, after screening for inclusion criteria, 231 records were excluded since they did not conducted in the hispanic cultural setting (211), and 20 for other reasons: not a study (description of a study protocol) (3) [25,26,27], education mixed with other interventions (8) [28,29,30,31,32,33,34,35], patients with LBP mixed with patients with other conditions (2) [36,37], not a randomized controlled trial (7) [36,38,39,40,41,42,43] and less than 10 patients per group (1) [44]; and nine RCTs were finally included in this review. Figure 1 shows the PRISMA flow diagram of this study.

Table 1 summarizes the main characteristics of the studies included in the systematic review. Two RCTs had randomized participants at the individual level [45,46], while the rest were cluster RCTs. Five studies were conducted with children, in the school setting [47,48,49,50,51], and four with adults; three in the clinical setting [18,45,46], and one in nursing homes [17].

Three studies, designed to assess the effectiveness of education as a treatment for LBP, were conducted with adult patients who had been recruited in the clinical setting [18,45,46]. Five studies assessing education for LBP prevention, included school children [47,48,49,50,51]. The ninth study assessed the potential effect of education for prevention and treatment, and was conducted with elderly living in nursing homes. This study included subjects both with and without LBP upon recruitment, and analyzed separately results for the whole sample and for participants who reported LBP [17].

Education on “active management” (i.e., primarily focusing on recommending avoiding bed rest and keeping as physically active as pain allowed to) was assessed in three studies [17,18,47], education on “postural hygiene” (i.e., primarily focusing on how to perform daily activities minimizing the load for the spine) in seven [17,18,45,46,48,49,51], education on exercise (i.e., teaching how to perform exercises) in four [18,45,46,50] and education on pain neurophysiology (aiming at altering patients’ knowledge about their pain states and conceptualizing pain) [52] in one [46]. Comparators were no intervention, usual care, exercise, other types of education (including short education programs on cardiovascular health and on weight control and healthy nutrition habits, which were considered “sham” educational interventions for LBP), and different combinations of these procedures.

The intensity and duration of the education programs varied widely across studies. In the clinical setting, it varied from a 20 min group talk and the handing out of a leaflet [17,18], to a 11 min video to be seen daily, 5 days a week for 9 months, combined with a face-to-face visit and as many contacts with the researchers as the participants wished during one year [45]. In the school setting, it ranged from handing out a comic book in class [47], to six one-hour sessions [48,51] or two 13 min sessions per week during 32 weeks [50].

In studies conducted with adults, outcomes across studies included LBP-related disability, pain severity (for LBP and referred pain down to the leg), 9 month LBP prevalence, health-related quality of life, fear avoidance beliefs (FABs), catastrophizing, kinesiophobia, finger to floor distance, pressure pain thresholds, and muscle endurance (Shirado–Ito abdominal and lumbar tests [53]). In studies conducted with children, outcomes were knowledge (on active management or postural hygiene), weight of the backpack, pain severity and 1 week LBP prevalence (Table 2).

Table 2 shows the main results of each study.

Table 3 shows the risk of bias of the studies included in this review. Five RCTs were categorized as “low risk of bias” [17,18,45,46,47]. Their results suggest that with regard to education programs designed for adults: (a) the combination of education on postural hygiene and exercise improves on results from usual care [45]; (b) education on pain neurophysiology improves the results of education on exercise [46]; (c) education on “active management” is more effective than education on postural hygiene [17], education on cardiovascular health [17], and on bodyweight control and heathy nutrition habits [18]; (d) adding a combination of education on postural hygiene and exercise does not significantly improve the results of education on active management [18]. With regard to education programs designed for children, the handing out of a comic book in class is effective to transmit knowledge on active management [47].

Four RCTs were categorized as “high risk of bias” [48,49,50,51]. All of them relate to education programs designed for school children, and their results suggest that (a) education in class is effective to transmit knowledge on postural hygiene [48,51], and to reduce the weight of their backpacks [49], and (b) education on exercise reduces the 1 week prevalence of LBP [50].

In all the RCTs with adults, education led to an improvement in LBP-related disability [17,18,45,46], which was above the cut-off value for clinical relevance [54,55]. Improvements in pain and quality of life were only reported in studies in which intensive programs involving exercise were implemented [45,46].

Several studies conducted with adults assessed the evolution of psychological variables after education (e.g., fear avoidance beliefs (FABs), catastrophizing, and kinesiophobia) [17,18,46]. All showed an improvement in these variables following education. Two studies analyzed the influence of the evolution of FABs and catastrophizing on disability, and showed that these psychological variables had no influence on the effect of education on disability [17,18].

## 4. Discussion

According to the results from this systematic review, education programs are effective for treating patients who suffer from LBP in the Hispanic cultural environment. All the studies including patients showed that those receiving any kind of education programs experienced an improvement in disability. Additionally, some studies in which exercise was also promoted reported improvements in pain and health-related quality of life. The effect sizes were generally small, but above the cut-off value for clinical relevance (Table 2) [54,55]. These results are generally consistent with those from studies conducted in other cultural settings [56,57]. In fact, the small size of the effect on disability triggered by education is in line with most medical treatments for LBP [6,7,58]

It is impossible to rule out that unspecific effects contributed to the outcomes following education. For instance, some education programs were intense, lasted up to one year and implied a frequent contact with therapists and researchers. All of this may have triggered powerful unspecific effects. Moreover, any education program, irrespective of its content, organization and approach, can have a psychological effect by making patients with LBP feel that they are better prepared to face daily activities, and potentially improve disability.

However, although unspecific effects may have magnified the impact of education in some studies, results from this study suggest that some types of education are likely to have an effect beyond unspecific effects. In fact, a significantly higher improvement in disability after education on active management, vs. postural hygiene, was observed in a study in which patients in both groups had the same interaction with therapists and researchers, received a comparable amount of attention, and all measures were taken to ensure that both patients and therapists were neutral with regard to both types of education (Table 1) [17].

Some studies assessed the evolution of psychological variables, namely FABs, catastrophizing and kinesiophobia, and found improvements after education [17,18,46]. However, those studies in which the influence of these variables on the improvement of pain or disability was explored, showed that such influence was non-existent [17,18]. This suggests that, in the Hispanic cultural environment, education simultaneously improves disability, FABs and catastrophizing, as opposed to the improvement of disability being mediated by the improvement of the latter.

Education programs might lead to deleterious consequences if they promoted misconceptions or inappropriate behavior. However, none of the studies with patients suffering from LBP recorded adverse events from the education programs. This may be because the authors assumed that the contents they were teaching were evidence based, and that the variables their studies gathered (e.g., disability, pain, health-related quality of life, and psychological variables) would have sufficed to capture any adverse events.

Very few medical treatments have been shown to have a clinically significant effect on LBP-related disability [6,7,58], which is the main cause of LBP-related social and economic burden [1,2,3]. Therefore, assuming that education did not lead to any significant adverse events, the fact that education programs improved LBP, and especially LBP-related disability, in the Hispanic environment, would support generalizing their use in clinical practice. This would require firstly defining which specific program or programs should be implemented.

Differences in methods and populations make it inappropriate to compare the effects of different types of education across studies. However, direct comparisons among different education programs within the same study are helpful to assess their comparative effectiveness. Cost, simplicity and amount of resources required by each education program are also likely to be essential for generalization in routine practice.

Therefore, future studies should compare the cost/effectiveness of the different education programs, assess their potential complementarity or summatory effects, and refine their indication criteria or implementation strategy.

Until these studies have been completed, the characteristics and results from the programs already implemented suggest that, among the different types of education which have been shown to be effective for adults suffering from LBP in the Hispanic environment, education on “active management” is the simplest. It requires a standardized 20 min group talk to groups of up to 20 patients, and the handing out of a specific leaflet (Table 1) [17,18]. This program has consistently been shown to be more effective than a program focusing on postural hygiene, both in middle-aged patients and elderly residents in nursing homes [17,18] (Table 2). This suggests that simple programs on active management might be appropriate as a first educational treatment in primary care and, if required, could be complemented at a later stage with more intensive and complex programs, involving prolonged exercise and education on pain neurophysiology [45,46].

In addition to the therapeutic effect of education for patients with LBP, several studies have assessed its potential application for primary prevention of LBP in the Hispanic environment. Due to the high prevalence of low back pain among the general population, and its increase with age [1,2,3], RCTs conducted outside the clinical environment require very large samples, long follow-up periods and low drop-out rates to detect a significant effect on LBP prevention. In fact, among the studies conducted in the school setting, only a low-quality study focused on the 1 week prevalence of LBP [50], while all the others focused on assessing whether the education programs where effective at transmitting the selected knowledge to the children [47,48,49,51]. This implies that these programs are only likely to be effective in practice if the concepts they transmit address proven risk factors or are actually effective at reducing the risk of LBP. Some evidence suggests that this is the case for exercise and active management [58,59,60,61,62,63], but not for backpack weight or form of carry [64,65].

### Limitations

This systematic review had some limitations. Despite a comprehensive search, only nine RCTs were identified, some were of low methodological quality and some gathered variables which are not clinically relevant. However, this limitation stemmed from the original studies included in this review, and five studies had a low risk of bias (four of which gathered clinically relevant variables), which made it possible for this review to draw conclusions and recommendations potentially useful for clinical practice.

Education on exercise was heterogeneous in terms of the specific exercises taught and the specific programs implemented. However, this is inherent to exercise in general, and the available evidence suggests that virtually any type of exercise is better than no exercise for both preventing and treating LBP [58,59,60,61,63].

Evidence on the effectiveness of education on pain neurophysiology and exercise, derived from only one study. However, evidence on education on active management is supported by several high-quality RCTs and, although this systematic review included only studies conducted in the Hispanic environment, results from studies conducted in other cultural settings are consistent [58,59,60,61,62,63].

All the RCTs which were identified as having taken place in the Hispanic cultural environment, had been conducted in Spain. Therefore, at this stage, it is unknown whether the conclusions from this review are applicable to the Hispanic populations living in South, Central or North America. This should be assessed in future studies.

## 5. Conclusions

In conclusion, this systematic review shows that the available evidence suggests that education on active management, exercise, and pain neurophysiology are effective for treating, and possibly preventing, LBP in the Hispanic cultural environment.

## Figures and Tables

**Table 1:** Main Characteristics of the Studies Included in the Systematic Review.

Study	Study Design	Setting	Follow-Up	N° of Subjects Included in the Analysis	Age (Years) ^1^	Intervention/s in the Experimental Group/s (EG)	Intervention/s in the Control Group (CG)	Statistical Analysis	Comments
**Kovacs et al., 2007** [17]	Cluster randomized trial	Nursing homes	6 months	N = 661 EG1 = 232 EG2 = 189 CG = 240	M(R) EG1 = 80.2 (77.0–83.1) EG2 = 81.6 (79.2–85.1) CG = 80.4 (76.5–83.4)	Usual care + 20 min talk, provided to groups of ≤20 participants, followed by hand out of a booklet (content consistent with the talk) Content: EG1 = Active management EG2 = Postural hygiene	Usual care + 20 min talk, provided to groups of ≤20 participants, followed by hand out of a booklet (content consistent with the talk) Content: Cardiovascular health	Mixed linear random-effects models	The same physician provided the education programs to all groups. He was told that the same effect was expected in both EGs, he had no opinion on their comparative effectiveness (both before and after the study) and was blind to subjects’ recruitment and assessment. An independent observer was present at the talks, and reported no differences across groups
**Albaladejo et al., 2010** [18]	Cluster randomized trial	Primary care	6 months	N = 348 EG1 = 215 EG2 = 139 CG = 171	M(IQR) EG1 = 51.0 (42.0; 58.0) EG2 = 51.0 (42.0; 59.7) CG = 52.5 (45.0; 61.7)	Usual care + EG1 and EG2: one 15 min talk on active management for low back pain, provided to groups of ≤20 participants, and handing out of a booklet with a consistent content EG2: + One additional 15 min talk, provided to groups of ≤20 participants, and handing out of a booklet on postural hygiene + Four 1 h/week sessions of physical therapy (exercise + stretching), in groups of ≤20 participants, and advice to continue at home	Usual care + one 15 min talk on the importance of weight control and healthy nutrition habits for the management of low back pain, provided to groups of ≤20 participants, and handing out of a booklet with a consistent content	Generalized estimating equations models	Subjects in the CG were told that weight control was very important for LBP
**Kovacs et al., 2011** [47]	Cluster randomized trial	School	98 days	N = 497 EG = 266 CG = 231	M(R) ^1^ 8 (7–9)	Handing out of a booklet on active management in class, adapted for 8-year-old children	No intervention	Intraclass correlation coefficient estimated by one-way ANOVA for the difference between scores (from baseline to end of follow-up)	
**Vidal et al., 2011** [48]	Cluster randomized trial	School	3 months	N = 137 EG = 63 CG = 74	M(SD) 10.72 (0.672) EG = 10.83 (0.636) CG = 10.64 (0.694)	6, one-hour sessions on postural hygiene + 4 talks on anatomy and physiology of the spine, pathophysiology of low back pain, risk factors, ergonomics, and postural hygiene + 2 “practical sessions”; postural analysis, carrying objects, balance, analysis of the content and form of carry for schoolbags, breathing and relaxation	No intervention	Repeated-measures analysis of co-variance (ANCOVA)	
**Del Pozo-Cruz et al., 2012** [45]	Randomized controlled trial	Primary care	9 months	N = 100 EG = 50 CG = 50	M(SD) EG = 45.50 (7.02) CC = 46.83 (9.13)	Usual care + face-to-face explanation of the program to each participant + As many contacts with researchers as participants wished (they could contact the research team by phone 5 days/week), with at least one face-to-face patient visit once a year + a website-based, educational program, including videos in which explanations were provided using audio and subtitles. Three videos were planned to be seen daily, from Monday to Friday, for 9 months: A 2 min video on postural hygiene at a computer workstation (ergonomically appropriate placement of the computer screen and the mouse pad, seat height, height of the armrest, etc.) A 7 min video on exercise (strengthening, flexibility, mobility and stretching, of abdominal, lumbar, hip and thigh muscles). Participants were asked not to perform any other physical exercise routine during the 9 month intervention period Viewing the same 2 min video on postural hygiene, once again + One reminder sent by e-mail (with instructions on how to access the Website), every day from Monday through Friday at 10 am, for 9 months	Usual care	Student’s *t* test for independent samples	
**Gallardo et al., 2013** [49]	Cluster randomized trial	School	3 months	N = 357 EG = 271 CG = 86	M(R) 8–10 (7–11) ^1^	One educational session on ergonomic criteria for selecting, loading and carrying a backpack (including the criterion to restrict carried items in the backpack to the minimum required). The content and distribution of items in the backpack on that very day, were analyzed	No intervention	Student’s *t* test	
**Rodríguez-García et al., 2013** [50]	Cluster randomized trial	School	8 months	N = 84 EG = 44 CG = 40	M(SD) ^1^ Children = 10.27 (0.31) Teenagers: 13.46 (0.68)	Education on physical exercise, in two 13 min sessions of exercise per week, during 32 weeks The exercises included hamstring stretching, endurance strength of abdominal and lumbar muscles, and anterior and posterior pelvic tilt	No intervention	Chi-square and Mann–Whitney U tests	
**Vidal et al., 2013** [51]	Cluster randomized trial	School	3 months	N = 137 EG = 63 CG = 74	M(SD) ^1^ 10.72 (0.672) EG = 10.83 (0.64) CG = 10.64(0.70)	6, one-hour sessions on postural hygiene 4 talks on anatomy and physiology of the spine, pathophysiology of low back pain, risk factors, ergonomics, and postural hygiene + 2 “practical sessions”; postural analysis, carrying objects, balance, analysis of the content and way to carry schoolbags, breathing and relaxation	No intervention	One-way analysis of variance (ANOVA) or chi-square tests, to compare baseline values. Repeated-measures analysis of co-variance (ANCOVA), to to examine the effect of the intervention	
**Bodes Pardo et al., 2018** [46]	RCT	Physical therapy practices	3 months	N = 56 EG = 28 EG = 28	M(SD) EG = 44.9(9.6); CG = 49.2(10.5)	Usual care + exercise (motor control exercises, stretching, and aerobic exercise): Session 1: exercises were demonstrated and performed by participants under supervision of a physiotherapist Session 2 (one month later). Same as session 1, confirming proper execution of exercises Patients instructed to complete the exercise program on their own, daily for 3 months. Compliance assessed + education on neurophysiology of pain (www.paininmotion.be) (accessed date: 29 September 2021). Two 30–50 min educational sessions on neurophysiology of pain, provided to groups of 4–6 participants Session 1: verbal explanation + visual presentation + leaflet reinforcing contents. Session 2, one month later: content of session 1 was reinforced, and questions answered	Usual care + exercise (motor control exercises, stretching, and aerobic exercise): Session 1: exercises were demonstrated and performed by participants under supervision of a physiotherapist. Session 2 (one month later). Same as session 1, confirming proper execution of exercises Patients instructed to complete the exercise program on their own, daily for 3 months. Compliance assessed	Pearson chi-square test and Student *t* test. Effect sizes were calculated by Hedges’ g	

RCT, randomized clinical trial; I, intervention; EG, experimental group; CG, control group; M(R): mean (range). M(IQR): mean (IQR: interquartile range). M(SD): Mean (Standard Deviation). ^1^ As per the Spanish law, children are grouped in class based on year of birth (e.g., all the children born between 1 January 2015 and 31 December 2015, are grouped in the same class). Therefore, the age of all the students in a school class is homogenous.

**Table 2:** Results.

Study	Outcome	Results (At the End of Follow-Up)	Comments
**Kovacs et al., 2007** [17] (CG = cardiovascular health; EG1 = active management; EG2 = postural hygiene) (see Table 1 for details)	Disability (RMDQ) Pain (VAS) Quality of life (PCS SF-12) Quality of life (MCS SF-12) Fear avoidance beliefs about physical activity (FAB-Phys),	Results at the cluster level. Results from mixed linear random-effects models: additional improvement over the control group [effect size (95%IC)]: EG1 = 1.0 (0.6, 3.4) EG2 = 1.1 (−0.5, 2.7) Change from baseline to the end of follow-up [mean (range)] EG1 = 3.3 (3.1–3.6) to 0.7 (0.3–1.1) EG2 = 3.5 (3.0- 4.0) to 1.2 (0.9–1.5) CG = 3.4 (3.3–3.5) to 1.4 (1.3;1.5) Change from baseline to the end of follow-up [mean (range)] EG1 = 35.8 (32.4–39.6) to 40.9 (37.7–44.3) EG2 = 31.7 (27.9–36.7) to 36.0 (34.5–38.4) CG = 34.4 (33.0–37.0) to 37.5 (36.5–39.3) Change from baseline to the end of follow-up [mean (range)] EG1 = 58.4 (57.4–59.3) to 58.9 (58.0–59.5) EG2 = 49.4 (45.0–52.9) to 57.7 (56.7–59.3) CG = 57.1 (57.0–57.2) to 58.6 (57.7–59.8) Change from baseline to the end of follow-up [mean (range)] EG1 = 16.9 (16.5–17.2) to 19.2 (18.8–19.6) EG2 = 19.4 (19.3–19.5) to 18.5 (18.4–18.7) CG = 18.9 (18.6–19.2) to 19.0 (18.8–19.1)	This study included subjects with and without low back pain when entering the study. Additional improvements of disability, over the CG, specifically in the subset of subjects who reported low back pain when entering the study, were: EG1: 3.0 (1.5, 4.5) EG2: 1.0 (−0.6, 2.7)
**Albaladejo et al., 2010** [18] (CG = weight control, EG1 = active management, EG2 = active management + postural hygiene + exercise supervised and unsupervised at home) (see Table 1 for details)	Disability (RMDQ) Low Back Pain (VAS) Referred pain (VAS) Catastrophizing (CSQ) Physical quality of life (PCS, SF12) Mental quality of life (MCS, SF12)	Results of the generalized estimating equations (GEE), adjusted for potential confounders, reflecting the improvement in each experimental group additional to the one in the control group [effect size (95% CI)] EG1 = 1.970 (1.252, 2.687) EG2 = 2.187 (1.413, 2.961) EG1 = 1.767 (1.363, 2.171) EG2 = 2.096 (1.660, 2.533) EG1 = 1.327 (0.831, 1.823) EG2 = 1.616 (1.055, 2.177) EG1 = 1.594 (0.659, 2.529) EG2 = 1.838 (0.834, 2.842) EG1 = 2.904 (1.256, 4.553) EG2 = 2.934 (1.163, 4.705) EG1 = 3.687 (1.711, 5.664) EG2 = 5.067 (2.933, 7.201)	
**Kovacs et al., 2011** [47] (CG = no intervention, EG = minimal intervention on active management) (see Table 1 for details)	Appropriate knowledge (scoring ≥ 80% of maximum possible correct responses in a questionnaire on back pain prevention and management)	Results of the generalized estimating equations (GEE), adjusted for potential confounders, reflecting the probability of “appropriate knowledge” in the EG over the CG [effect size (95% CI)] 1.61 (1.03–2.52)	
**Vidal et al., 2011** [48] (CG = no intervention, EG = intensive program on postural hygiene) (see Table 1 for details)	Healthy habits score (1 point given for each of the following items: “correct use of sofa”, “stooping correctly”, “taking care to sit correctly at home”, “taking care to sit correctly at school”, “frequent posture change on chair at home” and “frequent posture change on chair at school”) Range values: 0 (most “unhealthy” habits) to 6 (healthiest).	Results from a repeated-measures analysis of co-variance (ANCOVA) Comparison of scores before and after the intervention, showed a significant improvement in the EG (*p* < 0.001), but not in the CG (*p* > 0.6)	Actual scores in each group, are not disclosed (only graphically represented, separately for each of the items scored)
**Del Pozo-Cruz et al., 2012** [45] (CG = no intervention, EG = intense program on postural hygiene and exercise) (see Table 1 for details)	Disability RMDQ Number of episodes of LBP during the previous 9 months (i.e., prior to baseline vs. during the follow-up period) Quality of life (EQ-5D-3L, TTO score) Lumbar endurance test (Shirado–Ito test—measured in seconds) * Abdominal endurance test (Shirado–Ito test—measured in seconds) *	Change from baseline to post-intervention assessment (“intention to treat” analysis). Student’s *t* test for independent samples [mean ± standard deviation (95% CI)] EG: −6.76 ± 4.01 (−7.91, −5.61 *p* < 0.001 CG: 1.66 ± 2.59 (0.92, 2.39) *p* < 0.001 EG = −1.58 ± 6.73 (−1.77, −1.38) *p* < 0.001 CG = 0.18 ± 0.62 (0.001, 0.359) *p* = 0.048 EG = 0.20 ± 0.11 (0.17, 0.23) *p* < 0.001 CG = −0.01 ± 0.10 (−0.04, 0.01) *p* = 0.211 EG = 13.32 ± 26.58 (5.76, 20.87) *p* < 0.001 CG = −5.18 ± 20.19 (−10.91, 0.55) *p* = 0.076 EG = 13.98 ± 23.82 (7.20, 20.75) *p* < 0.001 CG = −4.66 ± 21.52 (−10.77, 1.45) *p* = 0.132	Only results from the “intention to treat” analysis are shown. Results from the “per protocol” analysis were consistent “*p*” values refer to intra-group differences (baseline values vs. value at the end of the 9 month, follow-up period)
**Gallardo et al., 2013** [49] (CG = no intervention, EG = one session on use of backpack) (see Table 1 for details)	Weight of the backpack (kg) (mean ± SD) Subjects carrying a backpack weighing <15% of bodyweight [n(%)]	Student’s *t* test At baseline: EG = 5.4 ± 1.5 CG = 5.9 ± 1.2 *p* = 0.011 Three months later: EG = 3.83 ± 1.47 CG = 5.89 ± 1.39 *p* < 0.001 At baseline: EG = 112 (41.3%) CG = 27 (31.4%) *p* = 0.100 Three months later: EG = 224 (82.7%) CG = 24 (27.0%) *p* < 0.001	Calculations based on the Number Needed to Treat (NNT) suggest that for every 100 children following the education program, 51 will reduce the weight of their backpacks to <15% of their bodyweight
**Rodríguez-García et al., 2013** [50] (CG = no intervention, EG = intensive program on exercise) (see Table 1 for details)	Number (%) of subjects reporting LBP in the previous week Pain severity	Student’s *t* test EG: Baseline: 8 (9.5%), 8 months follow-up: 2 (2.4%) CG: Baseline: 10 (11.9%) 8 months follow-up: 19 (22.6%) *p* < 0.05 No differences found (no data disclosed)	
**Vidal et al., 2013** [51] (CG = no intervention, EG = intensive program on postural hygiene) (see Table 1 for details)	“Healthy backpack use habits score” (1 point given for each of the following items: “try to load the minimum wight possible in the backpack”, “carry backpack on two shoulders”, “belief that backpack weight does not affect the back”, and “use of locker at school”). Range values: 0 (most “unhealthy” habits related to backpack) to 4 (healthiest).	Repeated-measures analysis of co-variance (ANCOVA): EG: the score improved at follow-up (*p* = 0.001) CG: no significant improvement in the score at follow-up (*p* = 0.2)	Actual scores in each group are not disclosed (only graphically represented, separately for each of the items scored)
**Bodes Pardo et al., 2018** [46] (CG = intensive program on exercise, supervised and unsupervised at home. EG = same program + education on neurophysiology of pain) (see Table 1 for details)	Disability (RMDQ), differences between scores at baseline and at 3 month follow-up pain (NPRS), differences between scores at baseline and at 3 month follow-up Physical quality of life (PCS, SF12), differences between scores at baseline and at 3 month follow-up Kinesiophobia (TSK-11), differences between scores at baseline and at 3 month follow-up Pressure pain thresholds (kg/cm^2^, using an analog Fisher algometer), differences between scores at baseline and at 3 month follow-up: On spinal process L3 On lateral epicondyle Finger to floor distance (cm), differences from baseline to 1 month follow-up Self-perception of improvement (PGIC)	Between-group difference in the variation of the score (Pearson chi-square or Student’s *t* test where appropriate) (mean (95%CI) −2.7 (−3.9, −1.4), *p* < 0.001 −2.2 (−2.93, −1.28), *p* < 0.001 −10.6 (−13.1, −8.06), *p* < 0.001 −8.5 (−11.0, −6.0), *p* < 0.001 1.21 (1.00, 1.41), *p* < 0.001 0.0 (−0.1, 0.01), *p* > 0.05 −2.6 (−4.5, −0.7), *p* < 0.05 *p* < 0.05	All differences were in favor of EG (a clinically positive change may imply a positive or negative score across variables, due to differences in the measuring instruments) Differences with regard to PGIC were reported in favor of the EG. However, actual values in each group were not provided; there were only graphically represented

EG, experimental group; CG, control group; CI, confidence interval. RMDQ: Roland–Morris Disability Questionnaire, ODI: Oswestry Low Back Pain Disability Index, VAS, visual analog scale, SF12: Spanish version of Short Form 12 (PCS; Physical Component Summary, MCS: Mental Component Summary), FAB-Phys: Fear Avoidance Beliefs on physical activity, CSQ: Coping Strategies Questionnaire, TSK: Spanish version of Tampa Scale for Kinesiophobia, PGIC: Patient Global Impression of Change, EQ-5D-3L (TTO): EuroQol-5Dimensions-3 Levels utility index (Time Trade-Off method). *: Shirado–Ito tests [53]. Scores ranges: RMDQ; 0 (no disability) to 24 (maximum disability). ODI: 0 (no disability) to 100 (maximum disability), VAS and NPRS: 0 (no pain) to 10 (worst imaginable pain). SF-12 PCS: 71.67 (best possible physical quality of life) to 2.86 (worst possible). SF-12 MCS: 71.24 (best possible mental quality of life) to 11.61 (worst possible). FAB-Phys: 0 (=no fear avoidance beliefs) to 30 (highest possible fear avoidance beliefs), CSQ: 0 = no catastrophizing, 36 = worst possible catastrophizing, EQ-5D-3L (TTO): 1 = best possible health-related quality of life (HRQL), 3 = worst possible HRQL. Shirado–Ito test; 120 s: best possible muscle endurance, 0 s: worst possible one. NPRS or NPRS11: Numeric Pain Rating Scale. TSK-11: 11 = no kinesiophobia, 44 = worst possible degree of kinesiophobia. PGIC: 7 (maximum possible self-perception of improvement) to 0 (minimum).

**Table 3:** Sources of Risk of Bias [24].

Bias Domain	Source of Bias	Studies								
		Kovacs et al. (2007) [17]	Albadalejo et al. (2010) [18]	Kovacs et al. (2011) [47]	Vidal et al. (2011) [48]	Del Pozo-Cruz et al. (2012) [45]	Gallardo Vidal et al. (2013) [49]	Rodriguez Garcia et al. (2013) [50]	Vidal et al. (2013) [51]	Bodes-Pardo et al. (2018) [46]
Selection	(1) Was the method of randomization adequate?	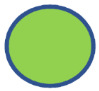	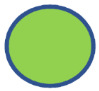	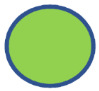	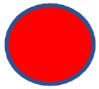	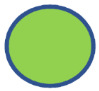	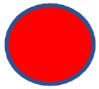	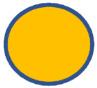	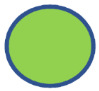	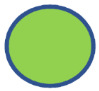
Selection	(2) Was treatment allocation concealed?	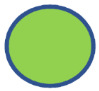	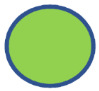	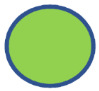	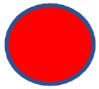	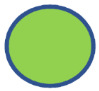	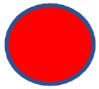	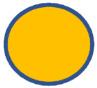	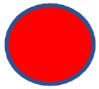	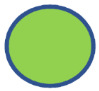
Performance	(3) Was the patient blinded to the intervention? ^1^	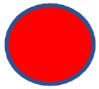	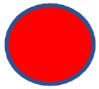	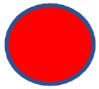	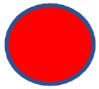	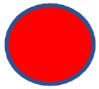	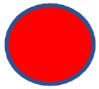	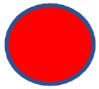	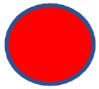	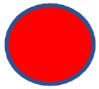
Performance	(4) Was the care provider blinded to the intervention? ^2^	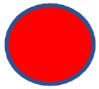	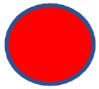	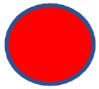	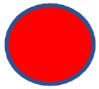	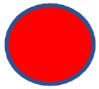	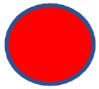	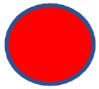	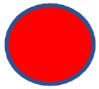	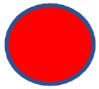
Detection	(5) Was the outcome assessor blinded to the intervention?	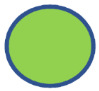	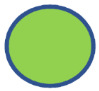	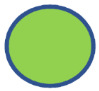	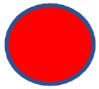	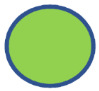	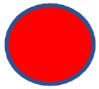	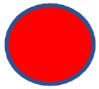	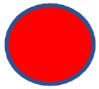	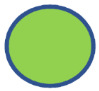
Attrition	(6) Was the drop-out rate described and acceptable?	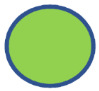	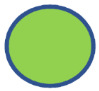	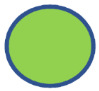	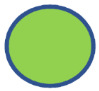	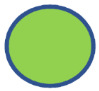	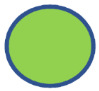	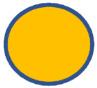	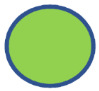	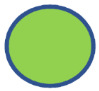
Attrition	(7) Were all randomized participants analyzed in the group which they were allocated?	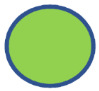	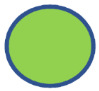	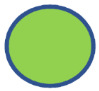	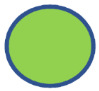	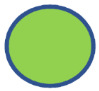	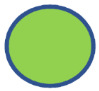	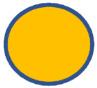	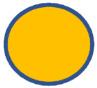	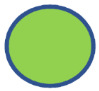
Reporting	(8) Are reports of the study free of suggestion of selective outcome reporting?	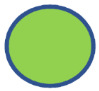	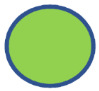	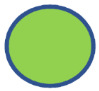	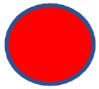	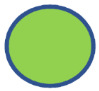	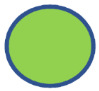	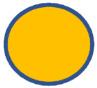	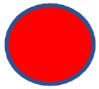	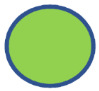
Selection	(9) Were the groups similar at baseline regarding the most important prognostic indicators, or were potential differences adjusted for at the analysis phase?	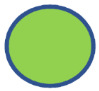	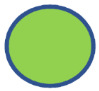	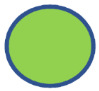	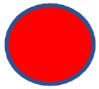	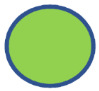	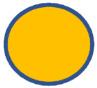	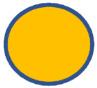	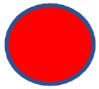	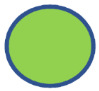
Performance	(10) Were cointerventions avoided or similar?	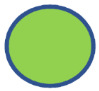	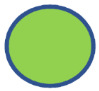	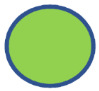	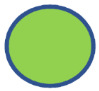	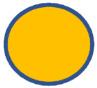	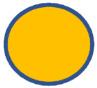	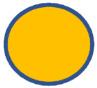	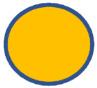	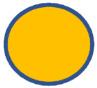
Performance	(11) Was the compliance acceptable in all groups?	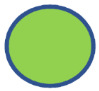	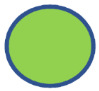	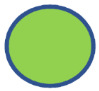	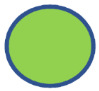	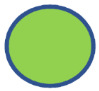	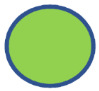	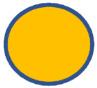	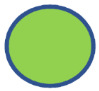	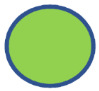
Detection	(12) Was the timing of the outcome assessment similar in all groups?	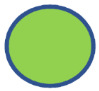	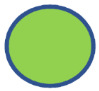	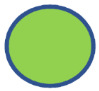	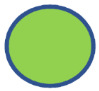	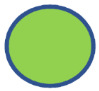	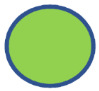	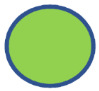	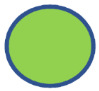	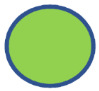
Other	(13) Are other sources of potential bias unlikely?	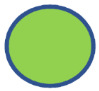	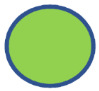	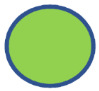	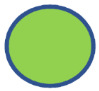	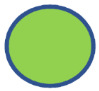	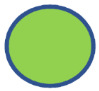	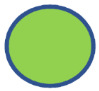	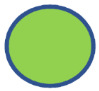	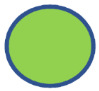
	**Total**	**11/13**	**11/13**	**11/13**	**06/13**	**10/13**	**06/13**	**02/13**	**05/13**	**10/13**

^1^: Because of the nature of the intervention, patients could not be blinded to whether they were receiving an intervention. However, in Kovacs 2007 and Albadalejo 2010, patients in the different groups received the same intervention; only the content of the education program was different, patients did not know that other groups were receiving different contents, and patients’ expectations were managed to be similar across groups. ^2^: Because of the nature of the intervention, the care provider could not be blinded. However, in Kovacs 2007 the care provider who gave the talks had no preferences on the content of the different education programs which were implemented in the control and the two experimental groups, either at the beginning and at the end of the trial, had been informed that the same outcome was to be expected across groups, and an independent physician audited that no differences in credibility or enthusiasm could be detected during the talks. Key, possible answers: Yes 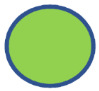 No 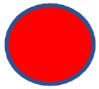 Unsure 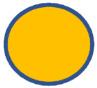.

## Data Availability

Not report any data.

## Appendix A

PUBMED

Search: (((((((low back pain) OR (back pain)) OR (back)) OR (musculoskeletal pain))) OR (musculoskeletal disorder)) OR (musculoskeletal diseases)) AND (((((((((education) OR (health-education)) OR (patient-education)) OR (therapeutic patient education)) OR (terapeutic patient education)) OR (physician health education)) OR (education intervention)) OR (medical community intervention)) OR (health community intervention)) Filters: Clinical Trial, Controlled Clinical Trial, Randomized Controlled Trial

((“low back pain”[MeSH Terms] OR (“low”[All Fields] AND “back”[All Fields] AND “pain”[All Fields]) OR “low back pain”[All Fields] OR (“back pain”[MeSH Terms] OR (“back”[All Fields] AND “pain”[All Fields]) OR “back pain”[All Fields]) OR (“back”[MeSH Terms] OR “back”[All Fields]) OR (“musculoskeletal pain”[MeSH Terms] OR (“musculoskeletal”[All Fields] AND “pain”[All Fields]) OR “musculoskeletal pain”[All Fields]) OR (“musculoskeletal diseases”[MeSH Terms] OR (“musculoskeletal”[All Fields] AND “diseases”[All Fields]) OR “musculoskeletal diseases”[All Fields] OR (“musculoskeletal”[All Fields] AND “disorder”[All Fields]) OR “musculoskeletal disorder”[All Fields]) OR (“musculoskeletal diseases”[MeSH Terms] OR (“musculoskeletal”[All Fields] AND “diseases”[All Fields]) OR “musculoskeletal diseases”[All Fields])) AND (“educability”[All Fields] OR “educable”[All Fields] OR “educates”[All Fields] OR “education”[MeSH Subheading] OR “education”[All Fields] OR “educational status”[MeSH Terms] OR (“educational”[All Fields] AND “status”[All Fields]) OR “educational status”[All Fields] OR “education”[MeSH Terms] OR “education s”[All Fields] OR “educational”[All Fields] OR “educative”[All Fields] OR “educator”[All Fields] OR “educator s”[All Fields] OR “educators”[All Fields] OR “teaching”[MeSH Terms] OR “teaching”[All Fields] OR “educate”[All Fields] OR “educated”[All Fields] OR “educating”[All Fields] OR “educations”[All Fields] OR (“health education”[MeSH Terms] OR (“health”[All Fields] AND “education”[All Fields]) OR “health education”[All Fields]) OR (“patient education handout”[Publication Type] OR “patient education as topic”[MeSH Terms] OR “patient education”[All Fields]) OR (“ther patient educ”[Journal] OR (“therapeutic”[All Fields] AND “patient”[All Fields] AND “education”[All Fields]) OR “therapeutic patient education”[All Fields]) OR (“terapeutic”[All Fields] AND (“patient education handout”[Publication Type] OR “patient education as topic”[MeSH Terms] OR “patient education”[All Fields])) OR ((“physician s”[All Fields] OR “physicians”[MeSH Terms] OR “physicians”[All Fields] OR “physician”[All Fields] OR “physicians s”[All Fields]) AND (“health education”[MeSH Terms] OR (“health”[All Fields] AND “education”[All Fields]) OR “health education”[All Fields])) OR ((“educability”[All Fields] OR “educable”[All Fields] OR “educates”[All Fields] OR “education”[MeSH Subheading] OR “education”[All Fields] OR “educational status”[MeSH Terms] OR (“educational”[All Fields] AND “status”[All Fields]) OR “educational status”[All Fields] OR “education”[MeSH Terms] OR “education s”[All Fields] OR “educational”[All Fields] OR “educative”[All Fields] OR “educator”[All Fields] OR “educator s”[All Fields] OR “educators”[All Fields] OR “teaching”[MeSH Terms] OR “teaching”[All Fields] OR “educate”[All Fields] OR “educated”[All Fields] OR “educating”[All Fields] OR “educations”[All Fields]) AND (“intervention s”[All Fields] OR “interventions”[All Fields] OR “interventive”[All Fields] OR “methods”[MeSH Terms] OR “methods”[All Fields] OR “intervention”[All Fields] OR “interventional”[All Fields])) OR ((“medic”[All Fields] OR “medical”[All Fields] OR “medicalization”[MeSH Terms] OR “medicalization”[All Fields] OR “medicalizations”[All Fields] OR “medicalize”[All Fields] OR “medicalized”[All Fields] OR “medicalizes”[All Fields] OR “medicalizing”[All Fields] OR “medically”[All Fields] OR “medicals”[All Fields] OR “medicated”[All Fields] OR “medication s”[All Fields] OR “medics”[All Fields] OR “pharmaceutical preparations”[MeSH Terms] OR (“pharmaceutical”[All Fields] AND “preparations”[All Fields]) OR “pharmaceutical preparations”[All Fields] OR “medication”[All Fields] OR “medications”[All Fields]) AND (“communal”[All Fields] OR “communalism”[All Fields] OR “communalities”[All Fields] OR “communality”[All Fields] OR “communally”[All Fields] OR “commune”[All Fields] OR “communes”[All Fields] OR “community s”[All Fields] OR “communitys”[All Fields] OR “residence characteristics”[MeSH Terms] OR (“residence”[All Fields] AND “characteristics”[All Fields]) OR “residence characteristics”[All Fields] OR “communities”[All Fields] OR “community”[All Fields]) AND (“intervention s”[All Fields] OR “interventions”[All Fields] OR “interventive”[All Fields] OR “methods”[MeSH Terms] OR “methods”[All Fields] OR “intervention”[All Fields] OR “interventional”[All Fields])) OR ((“public health”[MeSH Terms] OR (“public”[All Fields] AND “health”[All Fields]) OR “public health”[All Fields] OR (“health”[All Fields] AND “community”[All Fields]) OR “health community”[All Fields]) AND (“intervention s”[All Fields] OR “interventions”[All Fields] OR “interventive”[All Fields] OR “methods”[MeSH Terms] OR “methods”[All Fields] OR “intervention”[All Fields] OR “interventional”[All Fields])))) AND (clinicaltrial[Filter] OR controlledclinicaltrial[Filter] OR randomizedcontrolledtrial[Filter])

**
Translations
**

**
low back pain:** “low back pain”[MeSH Terms] OR (“low”[All Fields] AND “back”[All Fields] AND “pain”[All Fields]) OR “low back pain”[All Fields]

**
back pain:** “back pain”[MeSH Terms] OR (“back”[All Fields] AND “pain”[All Fields]) OR “back pain”[All Fields]

**
back:** “back”[MeSH Terms] OR “back”[All Fields]

**
musculoskeletal pain:** “musculoskeletal pain”[MeSH Terms] OR (“musculoskeletal”[All Fields] AND “pain”[All Fields]) OR “musculoskeletal pain”[All Fields]

**
musculoskeletal disorder:** “musculoskeletal diseases”[MeSH Terms] OR (“musculoskeletal”[All Fields] AND “diseases”[All Fields]) OR “musculoskeletal diseases”[All Fields] OR (“musculoskeletal”[All Fields] AND “disorder”[All Fields]) OR “musculoskeletal disorder”[All Fields]

**
musculoskeletal diseases:** “musculoskeletal diseases”[MeSH Terms] OR (“musculoskeletal”[All Fields] AND “diseases”[All Fields]) OR “musculoskeletal diseases”[All Fields]

**
education:** “educability”[All Fields] OR “educable”[All Fields] OR “educates”[All Fields] OR “education”[Subheading] OR “education”[All Fields] OR “educational status”[MeSH Terms] OR (“educational”[All Fields] AND “status”[All Fields]) OR “educational status”[All Fields] OR “education”[MeSH Terms] OR “education’s”[All Fields] OR “educational”[All Fields] OR “educative”[All Fields] OR “educator”[All Fields] OR “educator’s”[All Fields] OR “educators”[All Fields] OR “teaching”[MeSH Terms] OR “teaching”[All Fields] OR “educate”[All Fields] OR “educated”[All Fields] OR “educating”[All Fields] OR “educations”[All Fields]

**
health-education:** “health education”[MeSH Terms] OR (“health”[All Fields] AND “education”[All Fields]) OR “health education”[All Fields]

**
patient-education:** “patient education handout”[Publication Type]. or. “patient education as topic”[MeSH Terms]. or. “patient education”[All Fields]

**
therapeutic patient education:** “Ther Patient Educ”[Journal:__jid101517090] OR (“therapeutic”[All Fields] AND “patient”[All Fields] AND “education”[All Fields]) OR “therapeutic patient education”[All Fields]

**
patient education:** “patient education handout”[Publication Type]. or. “patient education as topic”[MeSH Terms]. or. “patient education”[All Fields]

**
physician:** “physician’s”[All Fields] OR “physicians”[MeSH Terms] OR “physicians”[All Fields] OR “physician”[All Fields] OR “physicians’s”[All Fields]

**
health education:** “health education”[MeSH Terms] OR (“health”[All Fields] AND “education”[All Fields]) OR “health education”[All Fields]

**
education:** “educability”[All Fields] OR “educable”[All Fields] OR “educates”[All Fields] OR “education”[Subheading] OR “education”[All Fields] OR “educational status”[MeSH Terms] OR (“educational”[All Fields] AND “status”[All Fields]) OR “educational status”[All Fields] OR “education”[MeSH Terms] OR “education’s”[All Fields] OR “educational”[All Fields] OR “educative”[All Fields] OR “educator”[All Fields] OR “educator’s”[All Fields] OR “educators”[All Fields] OR “teaching”[MeSH Terms] OR “teaching”[All Fields] OR “educate”[All Fields] OR “educated”[All Fields] OR “educating”[All Fields] OR “educations”[All Fields]

**
intervention:** “intervention’s”[All Fields] OR “interventions”[All Fields] OR “interventive”[All Fields] OR “methods”[MeSH Terms] OR “methods”[All Fields] OR “intervention”[All Fields] OR “interventional”[All Fields]

**
medical:** “medic”[All Fields] OR “medical”[All Fields] OR “medicalization”[MeSH Terms] OR “medicalization”[All Fields] OR “medicalizations”[All Fields] OR “medicalize”[All Fields] OR “medicalized”[All Fields] OR “medicalizes”[All Fields] OR “medicalizing”[All Fields] OR “medically”[All Fields] OR “medicals”[All Fields] OR “medicated”[All Fields] OR “medication’s”[All Fields] OR “medics”[All Fields] OR “pharmaceutical preparations”[MeSH Terms] OR (“pharmaceutical”[All Fields] AND “preparations”[All Fields]) OR “pharmaceutical preparations”[All Fields] OR “medication”[All Fields] OR “medications”[All Fields]

**
community:** “communal”[All Fields] OR “communalism”[All Fields] OR “communalities”[All Fields] OR “communality”[All Fields] OR “communally”[All Fields] OR “commune”[All Fields] OR “communes”[All Fields] OR “community’s”[All Fields] OR “communitys”[All Fields] OR “residence characteristics”[MeSH Terms] OR (“residence”[All Fields] AND “characteristics”[All Fields]) OR “residence characteristics”[All Fields] OR “communities”[All Fields] OR “community”[All Fields]

**
intervention:** “intervention’s”[All Fields] OR “interventions”[All Fields] OR “interventive”[All Fields] OR “methods”[MeSH Terms] OR “methods”[All Fields] OR “intervention”[All Fields] OR “interventional”[All Fields]

**
health community:** “public health”[MeSH Terms] OR (“public”[All Fields] AND “health”[All Fields]) OR “public health”[All Fields] OR (“health”[All Fields] AND “community”[All Fields]) OR “health, community”[All Fields]

**
intervention:** “intervention’s”[All Fields] OR “interventions”[All Fields] OR “interventive”[All Fields] OR “methods”[MeSH Terms] OR “methods”[All Fields] OR “intervention”[All Fields] OR “interventional”[All Fields]

Search: **(((((((low back pain[MeSH Terms]) OR (back pain[MeSH Terms])) OR (back[MeSH Terms])) OR (lumbago[MeSH Terms])) OR (musculoskeletal pain[MeSH Terms])) OR (musculoskeletal disorder[MeSH Terms])) OR (musculoskeletal diseases[MeSH Terms])) AND (((((((((education[MeSH Terms]) OR (health-education[MeSH Terms])) OR (patient-education[MeSH Terms])) OR (Therapeutic Patient Education[MeSH Terms])) OR (TPE[MeSH Terms])) OR (physician health education[MeSH Terms])) OR (educational intervention[MeSH Terms])) OR (medical community intervention[MeSH Terms])) OR (health community intervention[MeSH Terms])) Filters: Clinical Trial**

**MEDLINE**

TEMA: (low back pain OR back pain OR back OR lumbago OR musculoskeletal pain OR musculoskeletal disorder OR musculoskeletal diseases) *AND* TEMA: (education OR health-education OR patient-education OR Therapeutic Patient Education OR physician health education OR educational intervention OR medical community intervention OR health community intervention)

**Chocrane library**

**low back pain OR back pain OR back OR lumbago OR musculoskeletal pain OR musculoskeletal disorder OR musculoskeletal diseases in Title Abstract Keyword AND education OR health-education OR patient-education OR Therapeutic Patient Education OR physician health education OR educational intervention OR medical community intervention OR health community intervention in Title Abstract Keyword—(Word variations have been searched)**

**SCOPUS**

(TITLE-ABS-KEY (“low back pain” OR “back pain” OR back OR lumbago OR “musculoskeletal pain” OR “musculoskeletal disorder” OR “musculoskeletal diseases”) AND TITLE-ABS-KEY (education OR “health education” OR “patient education” OR “Therapeutic Patient Education” OR “physician health education” OR “educational intervention” OR “medical community intervention” OR “health community”))

**NHS CRD (DARE, HTA)**

(low back pain OR back pain OR back OR lumbago OR musculoskeletal pain OR musculoskeletal disorder OR musculoskeletal diseases) AND (Education OR health-education OR patient-education OR Therapeutic Patient Education OR physician health education OR educational intervention OR medical community intervention OR health community intervention) IN DARE, NHSEED, HTA

**CINAHL,**

TX (low back pain OR back pain OR back OR lumbago OR musculoskeletal pain OR musculoskeletal disorder OR musculoskeletal diseases) AND TX (Education OR health-education OR patient-education OR Therapeutic Patient Education OR physician health education OR educational intervention OR medical community intervention OR health community intervention)

**LILACS**

(Education OR health-education OR patient-education OR Therapeutic Patient Education OR physician health education OR educational intervention OR medical community intervention OR health community intervention) AND (low back pain OR back pain OR back OR lumbago OR musculoskeletal pain OR musculoskeletal disorder OR musculoskeletal diseases)

**SCIENCE DIRECT**

(low back pain OR back pain OR back OR lumbago OR musculoskeletal pain OR musculoskeletal disorder OR musculoskeletal diseases) AND (education)

SCIELO

Back AND Education

WEB OF SCIENCE

#8 AND #4

**Refinado por: PAÍSES/REGIONES:** (CUBA OR SPAIN OR COLOMBIA OR COSTA RICA OR FRANCE OR ECUADOR OR CHILE OR ARGENTINA OR VENEZUELA OR ESPANA OR URUGUAY OR BRASIL OR PERU OR MEXICO) AND **IDIOMAS:** (ENGLISH OR UNSPECIFIED OR SPANISH OR FRENCH) AND **TIPOS DE DOCUMENTOS:** (ARTICLE)

**Período de tiempo:** Todos los años. **Bases de datos:** WOS, CCC, DIIDW, KJD, MEDLINE, RSCI, SCIELO.

Idioma de búsqueda = Auto

**PEDRO**
